# Yellow Fever—Is the Cause Exotic or Indigenous?

**Published:** 1877-02

**Authors:** 


### YELLOW FEVER—IS THE CAUSE EXOTIC OR INDIGENOUS?

---

We have before called attention to this important subject, and now, after investigations have been had, must consider it in the light of facts presented. In the October number of the JOURNAL mention was made of the contradictory theories touching the cause of yellow fever, and the propriety of topographical examination of infected localities suggested. The opinion of its malarial origin was expressed in the November number, based on facts connected with this question, as obtained from various sources.

In regard to the various theories of yellow fever cause, we venture to assert that all may be reasonably reduced to two: one, the theory of contagion, the other non-contagion. We mean contagion in the generally accepted definition of the term, and not touching or innoculation, as the literal signification would imply. Small pox and measles are said to be communicated by contagion when the morbific material is received through the atmosphere, without contact. Thus it is that the near approach to the subject of these diseases is sufficient to communicate them, and the bedding or clothing of small pox patients, for days and even weeks after being thus used, retain the power of communicating the disease to others through the atmosphere, without contact. In this sense of contagion, then, we say that the exotic or importing theory of yellow fever cause, simply and clearly makes the disease contagious. The indigenous or local theory is founded on the supposed malarial origin.

We are in receipt of the annual report of the State Board of Health for 1876, in which is found a report "of the late epidemic of yellow fever in the State of Georgia." In this report are given the facts obtained by topographical inspection, and by the statements of those familiar with the infected cities and their surroundings, those who witnessed the prevalence of the disease, and those familiar with shipping from tropical regions to the ports of Georgia. Although the conclusion arrived at by the board, does not, from our standpoint, seem to be warranted by the facts, yet we are pleased to find them fully, and doubtless, fairly recorded. Not only so, but with becoming deference to the opinion of others, they advise the adoption of sanitary measures, which those, not agreeing with them in opinion as to cause, would rely upon for protection against the disease in future. For example, while they are clearly of the opinion that the cause is imported, and therefore the disease contagious in character, yet they earnestly advise the authorities to guard against the production of malaria, by preventing the overflow of marshy districts around Savannah, such as occurred during the warm season of last year.

In considering this subject we think too much importance cannot be given to the investigation of disease, when by doing so the cause may be subject to control. As suggested in a previous article, if the labor, time and money expended uselessly on quarantines, were used to prevent the known cause of malaria, thousands of valuable citizens might be saved to the State.

In the discussion of this subject we propose, first, to consider the question of contagion, personal infection or communication. We shall do so in the light of facts conceded by all. It is not contended that the so-called contagious diseases must necessarily attack all who come sufficiently near the subject, for some are more readily affected—having less constitutional power of resistance than others. In fact the same person's system may be in a more favorable condition at one time than at another to take on disease of any kind. We understand, however, that that disease which may be communicated from one person to another, will generally attack most of those coming within proper distance and not protected in some way against the morbific agent, at one locality as well as another. If malaria is necessary to the communication of vomito, of course it cannot be communicated without this poison, and the question is virtually decided that malaria produces the fever. The importationists are certainly driven to this dilemma; for no one familiar with the general known history of the disease, will deny that cases removed to a non-malarious locality do not communicate the disease to any, no matter how close the contact. This is proved by the report itself.

Not only is there no attempt to show an instance of communication in a non-malarious location, but the most thorough investigation and rigid examination of witnesses by the Board of Health while in Savannah, failed to prove that a case of yellow fever had been landed by any of the various vessels said to have brought the disease. At Brunswick, however, it was proven that Mrs. West actually went aboard the ship *Johnson*, where there were, or had been cases of the disease, and that she was attacked with yellow fever the next day. This struck us as a very short period of incubation, too short indeed for proof of contracting it from that source. In 1854, a gentleman was attacked with yellow fever in Atlanta, who had left Augusta, where the disease prevailed, a week or more before he was taken. Three cases came under our observation, also, in the interior of this State many years ago, who were said to have contracted the disease at Panama some weeks before.

The report of the Board of Health contains the statement of Army Surgeon, Dr. Woodhull and the attending physician, in regard to a case of yellow fever contracted on the Central Railroad some forty miles from Savannah, as was supposed, from sleeping in a car that came from the city where the disease was prevailing. This case is given as an instance, not of personal communication, but of transportation of Savannah malaria in the car. There is a discrepancy in the statements of the two physicians; one giving three, the other six days as the time of incubation. Both seem short, but as he had not been away from this healthy location to Savannah during the prevalance of fever, the conclusion must be that if the disease was true yellow fever, he contracted it from the car.

The information obtained from the Collector at Savannah and the United States Consul at Havana, in regard to the probable importation of yellow fever, is not at all favorable to that opinion. Although the facts of the disease prevailing at various places in Cuba, and of vessels passing from Cuban ports to Savannah, are given in the report as proof of it, yet the Consul states positively that no unusual amount of the disease existed at Havana or any other point on the island, while the Collector at Savannah gives a statement of the number of vessels arriving each month from tropical ports, without any startling revelation, calculated to convince any one that the contagion or importation theory is correct.

Taking a general view of the whole subject from the facts presented in the report it would seem that sailors landing at Savannah were probably the sufferers by it rather than our people, for no doubt many of them were exposed to the poison and doubtless contracted the disease while discharging ballast and loading their vessels with lumber and other commodities, near the marshes.

As to the question of malarial poison in the production of yellow fever, little need be said. With due deference to the conclusions arrived at by the Board, we must be permitted to say, that the facts presented in the evidence furnished, authorize the opinion that the fever in Savannah and Macon last summer was caused by marsh malaria. No doubt seems to be entertained of the existence of malaria in abundance, and of the prevalence of other forms of malarial fever beside yellow fever, such as remittent, intermittent, and perhaps hæmaturial. In Macon, those physicians most thoroughly impressed with the idea of importation, think that the malaria, not the disease, was imported; not entertaining the opinion of communication from a person having the disease.

This opinion of propagation by importation of cases of the fever is rejected by a very large proportion of observers during the endemic. Even this transportation of malaria from

Savannah to Macon in cars is stoutly denied by others, who found abundant evidences of malaria generated in the immediate vicinity of the Central Railroad depot, where the various forms of fever prevailed, and where, it was thought by one or two physicians, that the malarial poison was discharged from freight cars.

We shall refer to this subject again when certain facts are obtained, in regard to which we are now making thorough investigation.

